# Diagnoses, problems and healthcare interventions amongst older people with an unscheduled hospital admission who have concurrent mental health problems: a prevalence study

**DOI:** 10.1186/1471-2318-14-43

**Published:** 2014-04-02

**Authors:** Alex Glover, Lucy E Bradshaw, Nicola Watson, Emily Laithwaite, Sarah E Goldberg, Kathy H Whittamore, Rowan H Harwood

**Affiliations:** 1 Division of Rehabilitation and Ageing, University of Nottingham, Nottingham NG7 2UH, UK; 2 Health care of Older People, Nottingham University Hospitals NHS Trust, Queen’s Medical Centre, Nottingham NG7 2UH, UK

**Keywords:** Aged, Acute hospital, Diagnosis, Disability, Healthcare need, Mental health, Dementia, Delirium

## Abstract

**Background:**

Frail older people with mental health problems including delirium, dementia and depression are often admitted to general hospitals. However, hospital admission may cause distress, and can be associated with complications. Some commentators suggest that their healthcare needs could be better met elsewhere.

**Methods:**

We studied consecutive patients aged 70 or older admitted for emergency medical or trauma care to an 1800 bed general hospital which provided sole emergency medical and trauma services for its local population. Patients were screened for mental health problems, and those screening positive were invited to take part. 250 participants were recruited and a sub-sample of 53 patients was assessed by a geriatrician for diagnoses, impairments and disabilities, healthcare interventions and outstanding needs.

**Results:**

Median age was 86 years, median Mini-Mental State Examination score at admission was 16/30, and 45% had delirium. 19% lived in a care home prior to admission. All the patients were complex. A wide range of main admission diagnoses was recorded, and these were usually complicated by falls, immobility, pain, delirium, dehydration or incontinence. There was a median of six active diagnoses, and eight active problems. One quarter of problems was unexplained. A median of 13 interventions was recorded, and a median of a further four interventions suggested by the geriatrician. Those with more severe cognitive impairment had no less medical need.

**Conclusions:**

This patient group, admitted to hospital in the United Kingdom, had numerous healthcare problems, and by implication, extensive healthcare needs. Patients with simpler conditions were not identified, but may have already been rapidly discharged or redirected to non-hospital services by the time assessments were made. To meet the needs of this group outside the hospital would need considerable investment in medical, nursing, therapy and diagnostic facilities. In the meantime, acute hospitals should adapt to deliver comprehensive geriatric assessment, and provide for their mental health needs.

## Background

General hospitals may fail to meet all the needs of older people with co-morbid mental and behavioural disorders, including cognitive impairment, mood, alcohol problems and psychosis [1]. Two-thirds of older people in hospital have a mental disorder [2]. Twenty-five per cent of acute hospital beds accommodate someone with dementia, a proportion likely to rise given the ageing population and increasing prevalence of dementia [1,3]. A third of all patients admitted to general hospitals for acute care have delirium or dementia [4-7]. People with dementia are 1.4–3.6 times more likely to be admitted to hospital than age-matched controls [8,9]. Delirium is common in hospitalised older patients, especially those with dementia [10]. Both delirium and dementia are associated with excess mortality and the need for institutionalisation [4,6-8,11,12]. Much emphasis has been placed on mental health assessment and the provision of appropriate psychological and emotional care [2,13]. However, there has been little investigation of medical needs in this population.

Several large studies have looked at reasons for admission in people with dementia, basing their data on discharge or insurance records [8,9]. Pneumonia, urinary tract infections (UTI), falls and fractures are all common diagnoses [8,14-16]. It has been suggested that some patients are admitted unnecessarily, with conditions which could have been prevented or treated in primary care. These ‘ambulatory care sensitive conditions’ (ACSC) [17] may represent 40% of admissions of people with dementia [4]. Diagnoses reported to lead to admission are dependent on service configuration and data collection methods, and vary greatly between published studies. There is also a problem in assigning a single reason for admission in frail older adults, for whom comorbidity is common, and presentations often non-specific [18].

There is a debate about where frail older people who have combinations of problems affecting their physical and mental health should be treated [19]. With increasing emphasis on, and provision of, community services giving alternatives to hospital admission, the number and case mix of patients is likely to change over time, making up-to-date information important in providing services. We aimed to document diagnoses, problems, and healthcare interventions undergone by a sample of patients admitted to an acute general hospital who were identified as having a concurrent mental health diagnosis.

## Methods

### Study population

We recruited participants from two sites of an 1800-bed teaching hospital providing sole general medical and trauma services for a population of approximately 660,000 [5,20]. Consecutive patients aged over 70 with an unplanned admission lasting two or more days, were screened for inclusion if admitted to one of 12 wards, comprising two trauma orthopaedic, three acute geriatric medical and seven general medical wards. Those with a possible mental health diagnosis were identified by responses on the Abbreviated Mental Test score [21], four-point Geriatric Depression Score [22], CAGE alcoholism questions [23] and a question asking if there was any other reason to suspect a mental health diagnosis, and were invited to take part in the study. Written consent was taken from patients who had mental capacity; otherwise agreement was gained from a personal consultee (a family member or other individual, who, under English Mental Capacity law, can give agreement to a person taking part in research if they lack capacity). Fifty- three patients underwent a clinical assessment by a geriatrician. They were selected opportunistically, depending on availability of the research doctors, and all agreed to take part.

### Assessments

Participants, and a family carer where one was available and willing, were interviewed by a researcher, who also examined the case notes. They completed a battery of standardised health status measures, including admission problems, drugs, severity of medical illness (Modified Early Warning Score [24]), cognition (Mini-Mental State Examination, MMSE [25]), delirium (Delirium Rating Scale, DRS-R-98 [26]), mood (Cornell Scale for Depression in Dementia [27]), behavioural and psychological symptoms (Neuropsychiatric Inventory [28]), and physical disability (Barthel Index [29]).

Patients sub-sampled for diagnostic assessment were separately and independently assessed by one of three geriatricians, one consultant and two senior trainees. They were asked to complete a clinical assessment at the level expected for a thorough ward consultation, by examining case notes and investigations, talking to the patient and carers, and undertaking any further clinical examination required. No additional investigations were ordered. Proformas were completed detailing diagnoses, problems, social situation and contextual factors, interventions, including drugs stopped and started, and outstanding healthcare needs. Diagnoses were further classified as active, potentially active or inactive (at any time during the index admission), and level of diagnostic certainty as definite, probably or possible. Problems were defined clinically as issues considered important for the management of the case, and broadly represented risk factors, impairments or functional problems (activity limitations), and were qualified as explained or unexplained. Healthcare interventions undertaken by doctors, nurses and allied health professionals were noted if they were recorded in the case notes.

After hospital discharge professional coders routinely assigned a main admission diagnosis, working to a national coding manual, independently of clinicians and research assessments.

### Analysis

The population was described in terms of demographic features and measured health status. Diagnoses were coded according to the International Classification of Diseases 10th edition (ICD-10) [30], function using the International Classification of Functioning, Disability and Health (ICF) [31], and healthcare interventions using a bespoke hierarchical classification system. ACSC were identified using published criteria [17].

We calculated median numbers of diagnoses, problems and interventions, the prevalence of common diagnoses, and differences according to severity of cognitive impairment (MMSE score greater than 15 vs less than or equal to 15).

### Ethical approval

The study was approved by a research ethics committee approved for considering research on people lacking mental capacity (Nottingham 1 REC 08/H1302/127).

## Results

### Recruitment

Between April and November 2009 1004 patients were screened; 361 (36%) had no evidence of mental health disorder, 195 declined consent or consultee agreement, 61 had no contactable carer; 108 were excluded as they did not have capacity and we were unable to contact a carer prior to discharge; 8 were too ill and 21 were not recruited for other reasons. Two hundred and fifty were recruited and assessed at baseline for the main study. Fifty- three of these participants underwent a clinical assessment by a geriatrician. Median time after admission to geriatrician assessment was seven days (range 2–15, Inter Quartile Range, IQR, 6–10).

### Patient characteristics

Patients who were assessed by a geriatrician were similar to those who were not, apart from a greater proportion presenting with a fall (64% v 42%) and more having become immobile (74% v 58%; Table 1). One hundred and nine patients (44%) had an MMSE of 15 or less.

**Table 1:** Demographic variables and measured health status at admission

	**Assessed by geriatrician (n = 53)**	**Not assessed by geriatrician (n = 197)**
Median age/years (IQR)	86 (80–89)	84 (79–89)
Female	36 (68%)	129 (66%)
Geriatric medicine ward	26 (49%)	92 (47%)
General medicine ward	15 (28%)	70 (36%)
Orthopaedic ward	12 (23%)	34 (17%)
Care home residence	10 (19%)	42 (21%)
Median (IQR) Barthel ADL index prior to acute illness	17 (13–18.5)	16 (11–18)
Median (IQR) Barthel ADL index at admission	9 (4–14)	10 (5–14)
Median (IQR) cognitive function (MMSE)	16 (10–21)	17 (9–22)
Median (IQR) Cornell scale for depression in dementia	13 (8–17)	11 (7–15)
Median (IQR) Delirium Rating Scale	17 (10–24)	14 (7–23.5)
Categorical delirium (DRS > 18)	24 (45%)	83 (42%)
Mini-nutrition assessment - malnourished	18 (36%)	75 (38%)
Median (IQR) Charlson co-morbidity index	2 (1–4)	3 (1–4)
Median (IQR) number of medications	6 (4–9)	7 (5–9)
Median (IQR) total NPI score (behavioural and psychological symptoms)	24 (16–38)	24 (14–35)
Presentation with fall*	34 (64%)	82 (42%)
Presentation with reduced mobility*	39 (74%)	113 (58%)
Presentation with new incontinence	7 (13%)	25 (13%)
Presentation with current pressure sores	2 (4%)	9 (5%)
Presentation with dehydration	11 (21%)	20 (10%)
Presentation with worse cognition	22 (44%)	69 (35%)
Median length of stay in days (IQR)	14 (10–22)	12 (7–24)
Return to original place of residence	39 (75%)	140 (73%)

*P < 0.05 using Mann-Whitney or Chi squared tests.

ADL Activities of Daily Living.

NPI Neuropsychiatric Inventory.

### Coded admission diagnosis

The most common coded diagnosis among the whole cohort was fracture of the femur, followed by UTI, ‘senility’ and pneumonia (Table 2). UTI and pneumonia were more common in patients with lower MMSE scores; heart failure and chronic obstructive pulmonary disease (COPD) were less common.

**Table 2:** Commonest coded reasons for admission stratified by cognitive function

**ICD10 code**	**Diagnosis description**	**MMSE ≤15 (n = 109)**	**MMSE >15 (n = 140)**	**Total (n = 249)**
S72	Fracture of femur	16 (14.7%)	17 (12.1%)	33 (13.3%)
N39	Urinary tract infection	11 (10.1%)	7 (5.0%)	18 (7.2%)
R54	‘Senility’ (1)	8 (7.3%)	6 (4.3%)	14 (5.6%)
J18	Bronchopneumonia (2)	9 (8.3%)	3 (2.1%)	12 (4.8%)
I50	Heart failure	2 (1.8%)	7 (5.0%)	9 (3.6%)
J44	Chronic obstructive pulmonary disease (2)	1 (0.9%)	8 (5.7%)	9 (3.6%)
J22	Unspecified lower respiratory tract infection	3 (2.8%)	4 (2.9%)	7 (2.8%)
F01	Vascular dementia	3 (2.8%)	2 (1.4%)	5 (2.0%)
R55	Syncope	3 (2.8%)	2 (1.4%)	5 (2.0%)
S42	Fracture of clavicle	2 (1.8%)	3 (2.1%)	5 (2.0%)

MMSE Mini-Mental State Examination Score.

(1) a term not used in UK clinical practice, but appears in ICD-10 and, at the time, by clinical coders to express functional impairments, especially falls.

(2) p < 0.05 using Chi-squared or Fisher’s exact tests.

Thirty (12%) coded admission diagnoses were for ‘ambulatory care sensitive conditions’, and these were more frequent if MMSE ≤15 (relative risk 2.6, 95% CI 1.3–5.3).

### Geriatrician-assessed presenting diagnosis and problems

Following geriatrician assessment it was not always possible to assign a single main diagnosis. The main diagnoses were fractured neck of femur 7; other fractures 6; pneumonia 4; multifactorial fall 4; multifactorial functional problem 3; atrial fibrillation with fast ventricular response 3; dehydration/renal failure 3; UTI 1; alcohol intoxication 2; adverse drug reaction 2; seizures 2; unresponsive episode 2; painful hip post fall 2; unexplained delirium 2; cancer 2; exacerbation of COPD 1; infected leg ulcer 1; gastroenteritis 1; stroke 1; ruptured Achilles tendon 1; rheumatoid arthritis 1; progression of vascular dementia 1; acute urinary retention 1; anxiety 1.

Assessed presenting functional problems were immobility 38 (73%), falls 34 (64%), pain 28 (54%), incontinence 24 (46%), breathlessness 12 (23%), increased confusion 11 (21%), and dehydration 11 (21%).

### Multiple pathologies

The number of diagnoses per patient ranged from 5 to 19 with a median of 9 (IQR 6–10). Seventy-nine per cent were rated definite, 17% probable and 4% possible. 43% of diagnoses were active, 25% potentially active, and 31% inactive. This did not differ greatly by ward type (Table 3). The total number of active or potentially active diagnoses ranged from 2 to 16 with a median of 6 (IQR 5–8). The most common diagnoses were dementia, falls and musculoskeletal problems (Table 4). The only diagnosis significantly more common in patients with a low MMSE score was dementia.

**Table 3:** Median (minimum-maximum) number of assessed diagnoses and functional problems by ward type

		**Geriatric medical**	**General medical**	**Trauma Orthopaedic**	**Total**
Diagnoses	Any	9 (5–18)	8 (6–14)	10 (5–19)	9 (5–19)
	Active or potentially active	7 (2–13)	6 (3–11)	7 (3–16)	6 (2–16)
	Active	4 (1–9)	3 (1–7)	5 (2–10)	4 (1–10)
Problems	Recorded problems	10 (2–21)	7 (4–23)	8.5 (3–19)	9 (3–23)
	Impairments (ICF b-codes)	8 (2–17)	5 (3–21)	6.5 (2–17)	7 (2–21)
	Activity limitations (ICF d-codes)	2 (0–5)	2 (0–3)	2 (0–5)	2 (0–5)

**Table 4:** Assessed diagnoses stratified by cognitive function

**Diagnosis**	**MMSE ≤ 15 (n = 24)**	**MMSE > 15 (n = 29)**	**Total (n = 53)**
Dementia	22 (92%)	12 (41%)	34 (64%)
Fall/syncope^1^	13 (54%)	14 (48%)	27 (51%)
Arthritis and musculoskeletal pain^2^	9 (38%)	14 (48%)	23 (43%)
Fracture and injury^3^	8 (33%)	9 (31%)	17 (32%)
Urinary symptoms or incontinence^4^	9 (38%)	7 (24%)	16 (30%)
Depressive/anxiety disorder^5^	4 (17%)	8 (28%)	12 (23%)
Eye problems^6^	6 (25%)	6 (21%)	12 (23%)
Essential hypertension	4 (17%)	7 (24%)	11 (21%)
Delirium	7 (29%)	4 (14%)	11 (21%)
Osteoporosis/osteomalacia^7^	5 (21%)	6 (21%)	11 (21%)
Heart disease (apart from atrial fibrillation)^8^	3 (13%)	8 (28%)	11 (21%)
Faecal incontinence/constipation	6 (25%)	4 (14%)	10 (19%)
Chronic lung disease^9^	1 (4%)	8 (28%)	9 (17%)
Atrial fibrillation	5 (21%)	4 (14%)	9 (17%)
Acute/chronic kidney disease^10^	4 (17%)	5 (17%)	9 (17%)
Anaemia	6 (25%)	3 (10%)	9 (17%)
Urinary infection	5 (21%)	4 (14%)	9 (17%)
Deafness/vertigo	5 (21%)	3 (10%)	8 (15%)
Stroke/cerebrovascular disease	4 (17%)	3 (10%)	7 (13%)
‘Other’ nervous systems disorders^11^	1 (4%)	5 (17%)	6 (11%)
Peripheral arterial or venous disease^12^	2 (8%)	4 (14%)	6 (11%)
Drug side effects	4 (17%)	1 (3%)	5 (9%)
Malignancy^13^	0	5 (17%)	5 (9%)
Gastro-intestinal disorders^14^	2 (8%)	3 (10%)	5 (9%)
Diabetes	1 (4%)	4 (14%)	5 (9%)
Mental and behavioural disorders due to alcohol	0	4 (14%)	4 (8%)
Chest infection	2 (8%)	2 (7%)	4 (8%)
Presence of functional implants	1 (4%)	2 (7%)	3 (6%)
High cholesterol	2 (8%)	1 (3%)	3 (6%)

Prevalence for diagnoses recorded in at least three cases.

MMSE Mini mental State Examination.

1-3 participants had 2 codes corresponding to fall or syncope.

2-4 participants had 2 or 3 codes corresponding to arthritis and musculoskeletal pain.

3-4 participants had 2 or 3 codes corresponding to fracture/injury.

4-2 participants had 2 or 3 codes corresponding to urinary symptoms.

5-3 participants had 2 codes corresponding to depression or anxiety disorders.

6-2 participants had 2 or 3 codes corresponding to eye problems.

7-1 participant had 2 codes corresponding to osteoporosis/osteomalacia.

8-5 participants had 2 or more codes corresponding to heart disease.

9-1 participant had 2 codes corresponding to chronic lung disease.

10-3 participants had 2 codes corresponding to kidney disease.

11-3 participants had 2 codes corresponding to ‘other’ nervous system disorders.

12-1 participant had 2 codes corresponding to peripheral arterial or venous disease.

13-2 participants had 2 codes corresponding to malignancy.

14-1 participant had 2 codes corresponding to gastrointestinal disorders.

### Problems

Five hundred and seventeen problems were recorded; 418 were abnormalities of body function (ICF b-codes), 5 abnormalities of body structure (s-codes) and 94 activity limitations (d-codes). The median number per patient was 9 (range 5–19). Functional problems were more common on geriatric and orthopaedic wards than general medical wards (median 10 v 7; Table 3). Seventy-five per cent of problems were explained: 89% on geriatric medical, 69% on orthopaedic, 63% on general medical wards. The most common impairments were in cognition (70%), walking (70%), postural stability (58%), transfers (55%), pain (55%) and continence (49%; Table 5). Cognitive impairment, apathy, urinary and faecal incontinence were more common in those with a lower MMSE score.

**Table 5:** Assessed functional impairments

**ICF code**	**MMSE ≤15 (n = 24)**	**MMSE > 15 (n = 29)**	**Total (n = 53)**	**Functional problem**
b117	22 (92%)	15 (52%)	37 (70%)	Intellectual functions
d450	17 (71%)	20 (69%)	37 (70%)	Walking
b755	15 (63%)	16 (55%)	31 (58%)	Falls/postural stability
d420	11 (46%)	18 (62%)	29 (55%)	Transferring oneself
b280	12 (50%)	17 (59%)	29 (55%)	Pain
b620	17 (71%)	9 (31%)	26 (49%)	Urination functions
b525	12 (50%)	12 (41%)	24 (45%)	Defecation functions
b130	11 (46%)	9 (31%)	20 (38%)	Energy and drive
b430	5 (21%)	13 (45%)	18 (34%)	Lifting and carrying
b545	4 (17%)	9 (31%)	13 (25%)	Water, mineral and electrolyte balance
d599	5 (21%)	6 (21%)	11 (21%)	Other digestive, metabolic and endocrine
b152	3 (13%)	8 (28%)	11 (21%)	Emotional functions
b460	2 (8%)	9 (31%)	11 (21%)	Thought functions
b435	4 (17%)	5 (17%)	9 (17%)	Immunological system
b730	4 (17%)	5 (17%)	9 (17%)	Muscle power
b530	2 (8%)	7 (24%)	9 (17%)	Weight maintenance
b210	5 (21%)	4 (14%)	9 (17%)	Seeing
b230	4 (17%)	5 (17%)	9 (17%)	Hearing
b110	2 (8%)	6 (21%)	8 (15%)	Consciousness functions
b147	4 (17%)	4 (14%)	8 (15%)	Psychomotor functions
b429	1 (4%)	5 (17%)	6 (11%)	Other cardiovascular functions
b510	3 (13%)	3 (10%)	6 (11%)	Ingestion functions
b420	0	6 (21%)	6 (11%)	Blood pressure functions
b550	3 (13%)	3 (10%)	6 (11%)	Thermoregulatory function
B410	2 (8%)	4 (14%)	6 (11%)	Heart functions
B134	4 (17%)	2 (7%)	6 (11%)	Sleep functions
B455	0	6 (21%)	6 (11%)	Exercise tolerance functions
B610	3 (13%)	3 (10%)	6 (11%)	Renal function

Prevalence for impairments recorded in at least 10% of the population. Note that in ICF, problems are classified according to the positive function, rather than the deficit.

MMSE Mini mental State Examination.

ICF International Classification of Functioning, Disability and Health.

### Interventions

In total 727 interventions were recorded (Table 6). Patients received a median of 13 recorded interventions (range 6–30) with a further four (range 0–26) recommended following geriatrician assessment. Twenty-five patients had medications stopped (mean 2.2 per patient), and 40 patients had new medications started (mean 2.8 per patient).

**Table 6:** Interventions

		**Documented in casenotes**	**Additional interventions suggested by geriatrician**
Assessment	Any	53 (100%)	36 (68%)
	Investigations	42 (79%)	17 (32%)
	Function	30 (57%)	7 (13%)
	Risk assessment	25 (47%)	0
	Collateral history	13 (25%)	17 (32%)
	Examination	53 (100%)	14 (26%)
	Use of standardised scales	1 (2%)	10 (19%)
	Diagnosis	53 (100%)	3 (6%)
Personal maintenance	Basic activities of daily living assistance	42 (79%)	2 (4%)
Therapy	Any	53 (100%)	40 (75%)
	Physiotherapy	39 (74%)	21 (40%)
	New drug prescription	36 (68%)	18 (34%)
	Drug review	21 (40%)	7 (13%)
	Intravenous fluid	17 (32%)	2 (4%)
	Oxygen	5 (9%)	0
	Occupational therapy	8 (15%)	7 (13%)
	Dietetics	6 (11%)	7 (13%)
	Surgery	8 (15%)	0
	Skin intervention	6 (11%)	1 (2%)
Monitoring	Any	53 (100%)	9 (17%)
Information giving	Any	19 (36%)	10 (19%)
	Discussion with family	16 (30%)	9 (17%)
Planning	Any	18 (34%)	20 (38%)
	Discharge planning	13 (25%)	12 (23%)

## Discussion

### Summary of findings

This study found that the main diagnosis assessed by a geriatrician as causing the hospital admission was varied, and sometimes difficult to assign. Musculoskeletal problems and dementia were very common. Functional problems and complications such as falls, immobility, incontinence and delirium were almost universal, and often multiple. Other than the preponderance of fracture and injury on trauma orthopaedic wards prevalence of both diagnoses and problems did not vary greatly by ward type. Some differences in diagnoses were evident in the group with the most severe cognitive impairment, including a higher proportion categorised as ‘ambulatory care sensitive conditions’, but if anything this group had more, not fewer, functional problems. A wide range of multidisciplinary interventions was delivered in hospital, and further interventions were recommended following assessment by a geriatrician, including starting and stopping medication, nursing and therapist interventions, family discussions, and follow up. Routine coding often differed from that recorded after geriatrician assessment.

### Strengths and weaknesses

A strength of this study is that it was based on comprehensive assessment rather than coded discharge diagnoses or insurance records and therefore was able to capture the complexity of the patient cohort. The study was conducted in a single UK National Health Service Hospital Trust, which provided sole emergency medical services for its local population. For practical reasons we recruited from only three of five geriatric medical wards, seven of eleven general medical wards, and two of three trauma orthopaedic wards. The study was relatively small, since assessments were time consuming. We did not recruit from specialist stroke, renal, neurology, cardiology, haematology, oncology or infectious diseases wards. The particular local configuration of these services will have influenced case mix, and limits generalisability, although we attempted to make the study as representative of ‘unselected’ general medical, geriatric and trauma cases as is possible in a modern health service.

Assessments were made about one week into the admission, by which point approximately one-third of participants recruited to the study had been discharged [5]. The time delay allowed an overview of sometimes fast-moving and fluid diagnostic formulations, but patients with simpler or transient problems amenable to alternative care outside the hospital will have been excluded, and trauma and falls were over-represented. We also excluded from recruitment those thought imminently likely to die.

Data predated the introduction of a routine orthogeriatric liaison service. Assessments were made by experienced geriatricians on the basis of clinical opinion, but research diagnostic criteria were not used. Diagnoses and problems were considered important to managing the case, but it was difficult to ascertain all problems without a direct functional assessment, nor to describe problems and interventions to a consistent degree of detail. For example, mobility problems might be broken down into bed mobility, transfers, and walking. Much routine nursing activity was not recorded, such as encouragement to eat or help using the toilet. Complex chains of diagnoses, consequences and complications could arise; for example, pneumonia may be complicated by delirium and a fall which results in a fracture. Any of these might be considered the ‘main’ diagnosis. ICD-10 is mostly a classification of pathologies, but includes some ‘functional diagnoses’ such as falls or incontinence, which also appear in ICF. Classification to one of these diagnoses sometimes depends on whether an alternative pathological diagnosis was available, or whether the problems had been identified and recorded in case notes, which can be inconsistent. For most participants, but not all, a mental health diagnosis was identified by the geriatrician. Those that did not may have recovered from delirium, or have been a false positive on initial screening, for example, due to the overlap between the effects of physical and mental illness. No inter-rater reliability testing was undertaken.

### Interpretation and context

It has been suggested that many patients with dementia are admitted with conditions that could have been prevented or managed outside the hospital [19]. This was confirmed by this study according to proposed criteria: ‘conditions for which hospital admission could be prevented by interventions in primary care’ [17]. Typically, however, older people present with non-specific functional problems such as immobility or falls which may be contributed to by several diagnoses, and the functional problems drive admissions rather than individual diagnoses. Functional decline creates acute dependency (sudden need for increased human help) and makes discharge difficult [32]. Patients with memory problems are likely to struggle in proactively managing their own medical conditions. Structured interviews with providers of hospital and community services for older patients have identified ‘internal’ and ‘external’ factors triggering hospital admission. Internal factors included features such as stoicism and reluctance to seek early medical help. External factors included access to alternative provision which tended to be complex making it difficult for patients to know where to seek help [33]. Proactive services such as community matrons (specialist community nurses) can help meet this medical need [34], but identifying all those at risk of hospital admission, and intervening successfully to prevent it, remains difficult [35,36].

Our findings are not consistent with the suggestion that large numbers of older adults with mental health problems are being admitted with little medical need. In the geographical area and hospital studied this may indicate that systems to avoid unnecessary admission and to enable early discharge were operating successfully. A variety of alternative models to hospital inpatient care has been described, including short stay assessment units, and intermediate care [37-41]. This study was unable to assess if length of hospital stay was appropriate or justified, or if care could have been provided in other settings. But the prevalence of impairments such as immobility, falls, delirium and incontinence implies the need for skilled nursing and rehabilitation, and the number of active medical problems suggests the need for medical diagnosis and management.

The tendency of older patients to present with non-specific and functional problems, to have multiple pathologies, to be prone to complications, lose abilities quickly when ill, and need explicit rehabilitation is well established [42]. Hospitalisation allows for comprehensive geriatric assessment (CGA), which high quality evidence demonstrates increases the chances of survival and regaining independence following illness [43,44].

### Future work

Our findings suggest that alternatives to hospital must have access to clinicians specialising in geriatric medicine and psychiatry, access to diagnostic technology, and the provision of intensive skilled nursing and rehabilitation. This level of care may be difficult to achieve cost-effectively outside of traditional hospitals. If this is the case, hospitals should be reformed to make them better able to meet the needs of elderly service users. For example, systems are needed to provide comprehensive geriatric assessment and multi-disciplinary management, there should be adequate provision of expert mental health care, and the environment and procedures need to be adapted for those with sensory or cognitive impairment. Rehabilitation and end of live care are required as well as acute medical or surgical care. Provision should be made for close communication and working with families and other informal carers [1,2,43-50]. Work is needed to define the limits of achievable care provision out of hospital, and to improve both outcomes and experiences of hospital care for frail older people. The study also suggests that routine diagnostic coding and information systems need to change to describe frail elderly patients better, in particular to include non-specific presentations and functional information, if they are to serve the needs of service planning and evaluation.

## Conclusions

Older people who are admitted to a general hospital and found to have co-morbid mental health problems also had many physical diagnoses and functional problems, and by implication extensive healthcare needs. Patients with simpler ‘ambulatory sensitive’ conditions were not identified, but may have already been rapidly discharged or redirected to non-hospital services by the time assessments were made. To meet the needs of this group outside the hospital would need considerable investment in medical, nursing, therapy and diagnostic facilities. In the meantime, acute hospitals (and alternative facilities) should adapt to make their environment and systems more appropriate for frail older people, to deliver comprehensive geriatric assessment, and provide for their mental health needs.

## Abbreviations

ACSC: Ambulatory care sensitive condition; CAGE: (mnemonic) screening tool for alcoholism; CGA: Comprehensive geriatric assessment; COPD: Chronic obstructive pulmonary disease; DRS-R-98: Delirium Rating Scale; ICD-10: International Classification of Diseases 10th edition; ICF: International Classification of Functioning, Disability and Health; IQR: Inter Quartile Range; MMSE: Mini-Mental State Examination; NPI: Neuropsychiatric inventory; UTI: Urinary tract infection.

## Competing interests

The authors declare that they have no competing interests.

## Authors’ contributions

Conception and design: RH; literature review AG, RH; recruitment and data collection SG, KW, NW, EL, RH; analysis LB, AG, RH, KW; interpretation, drafting of paper RH, AG; all authors contributed to editing and approved the final text.

## Pre-publication history

The pre-publication history for this paper can be accessed here:

http://www.biomedcentral.com/1471-2318/14/43/prepub
